# Media Use Behavior Mediates the Association Between Family Health and Intention to Use Mobile Health Devices Among Older Adults: Cross-Sectional Study

**DOI:** 10.2196/50012

**Published:** 2024-02-19

**Authors:** Jinghui Chang, Yanshan Mai, Dayi Zhang, Xixi Yang, Anqi Li, Wende Yan, Yibo Wu, Jiangyun Chen

**Affiliations:** 1 School of Health Management Southern Medical University Guangzhou China; 2 School of Public Health Southern Medical University Guangzhou China; 3 School of Public Health Peking University Beijing China

**Keywords:** older adults, family health, media use, intention to use mobile health devices, China

## Abstract

**Background:**

With the advent of a new era for health and medical treatment, characterized by the integration of mobile technology, a significant digital divide has surfaced, particularly in the engagement of older individuals with mobile health (mHealth). The health of a family is intricately connected to the well-being of its members, and the use of media plays a crucial role in facilitating mHealth care. Therefore, it is important to examine the mediating role of media use behavior in the connection between the family health of older individuals and their inclination to use mHealth devices.

**Objective:**

This study aims to investigate the impact of family health and media use behavior on the intention of older individuals to use mHealth devices in China. The study aims to delve into the intricate dynamics to determine whether media use behavior serves as a mediator in the relationship between family health and the intention to use mHealth devices among older adults. The ultimate goal is to offer well-founded and practical recommendations to assist older individuals in overcoming the digital divide.

**Methods:**

The study used data from 3712 individuals aged 60 and above, sourced from the 2022 Psychology and Behavior Investigation of Chinese Residents study. Linear regression models were used to assess the relationships between family health, media use behavior, and the intention to use mHealth devices. To investigate the mediating role of media use behavior, we used the Sobel-Goodman Mediation Test. This analysis focused on the connection between 4 dimensions of family health and the intention to use mHealth devices.

**Results:**

A positive correlation was observed among family health, media use behavior, and the intention to use mHealth devices (r=0.077-0.178, P<.001). Notably, media use behavior was identified as a partial mediator in the relationship between the overall score of family health and the intention to use mHealth devices, as indicated by the Sobel test (z=5.451, P<.001). Subgroup analysis further indicated that a complete mediating effect was observed specifically between family health resources and the intention to use mHealth devices in older individuals with varying education levels.

**Conclusions:**

The study revealed the significance of family health and media use behavior in motivating older adults to adopt mHealth devices. Media use behavior was identified as a mediator in the connection between family health and the intention to use mHealth devices, with more intricate dynamics observed among older adults with lower education levels. Going forward, the critical role of home health resources must be maximized, such as initiatives to develop digital education tailored for older adults and the creation of media products specifically designed for them. These measures aim to alleviate technological challenges associated with using media devices among older adults, ultimately bolstering their inclination to adopt mHealth devices.

## Introduction

### Background

The 2022 United Nations report on “World Population Prospects” predicted that by 2050, the global population will reach 9.7 billion. Within this demographic shift, 1.5 billion individuals aged 65 and above are anticipated, constituting 16% of the total population [1]. Notably, the trend of population aging is intensifying. In the context of population dynamics, China, as a heavily populated nation, is undergoing significant and intricate transformations. The Seventh National Population Census of China revealed that there are 264 million individuals aged 60 or older in the country, comprising 18.7% of the overall population [2]. This underscores the profound changes in China’s demographic landscape. The rapidly increasing aging rate in China poses substantial challenges for the future development of the country’s medical services. Over 180 million older adults in China grapple with chronic diseases, and a staggering 75% of them contend with multiple chronic illnesses [3]. This places older individuals in a high-risk and vulnerable category, imposing considerable financial and operational burdens on China’s medical and health sector.

Mobile health (mHealth) devices typically encompass mHealth programs and wearable devices [4]. Functioning as portable tools leveraging internet communication technology, these devices continuously monitor diverse physiological conditions. They have the capability to track and record users’ daily lifestyle and health status data in real-time [5]. These real-time data are instrumental for users to make informed adjustments to their health behaviors, facilitated by prompt feedback on health information [6]. The utilization of mHealth devices addresses the emerging need for self-monitoring and self-management within the expanding medical service market, aligning with heightened health awareness among consumers. These devices play a pivotal role in enabling early diagnosis, intervention, clinical treatment, and monitoring of various diseases by continuously supervising vital signs in real-time. However, it is noteworthy that despite the potential benefits, mHealth devices are not widely embraced by older individuals [7]. Consequently, the robust functionalities and inherent advantages of these devices remain underutilized within this demographic group. Emerging as an inevitable outcome of the internet era and the aging society, mHealth holds substantial potential to offer a promising solution to meet the escalating demands for medical services in developing countries [8]. Recognizing that older individuals constitute the most frequent and substantial users of health services [9], it becomes imperative to cultivate a new social trend, encouraging the integration of older individuals with mHealth [10].

Prior research has demonstrated that mHealth can significantly enhance the health, well-being, and longevity of older individuals in the digital era. However, it also introduces a new social governance challenge—the digital divide among older individuals [11,12]. This divide arises from challenges in accessing or utilizing information infrastructure coupled with a lower level of digital education, resulting in difficulties for older individuals to stay abreast of social, economic, and technological advancements [13]. As outlined in the 50th Statistical Report on the Development of the Internet in China by the China Internet Network Information Center, individuals aged 60 and above constitute the predominant group of non-netizens, comprising 41.6% of this demographic [14]. A confluence of personal, family, social, and technological factors collectively contributes to the estrangement of older individuals from engaging with new media, such as the internet [15]. Research indicates that the motivation for older individuals to actively seek health information on the internet is closely tied to their interactions with family or friends [16]. Older adults primarily rely on their families for social support, and the cohesion within the family unit significantly influences their overall health status [17,18].

Family health represents a collective resource that emerges from the interconnected well-being of each family member, encompassing their health, interactions, capacities, and the family’s overall physical, social, emotional, economic, and medical resources [19]. As an interdisciplinary concept, evaluating family health necessitates a thorough examination of various factors, including but not limited to family functioning, emotional support, financial resources, and access to external services [20]. Existing literature demonstrates that family support plays a pivotal role in motivating older individuals to seek medical services [21]. Additionally, family function and overall health serve as crucial indicators for assessing the mental well-being of older individuals [22]. Communication within the family, involving interactions with children, grandchildren, and peer groups, influences older individuals’ inclination to adopt smart senior care solutions [23]. While numerous articles predominantly explore family health from a singular dimension [24-26], there exists a research gap concerning the specific influence of family health on older individuals’ intention to adopt mHealth devices.

The evolution of mHealth is intricately linked to the technical backing of media. Media technology plays a dual role—it not only generates visual data representing health conditions detected by mHealth devices [27] but also serves as a platform for the public to exchange and share medical information. In the case of older adults, their acceptance of new health services and access to health information are influenced in distinct ways by the utilization of media devices [28,29]. A Chinese empirical analysis revealed a fundamental correlation between media use and the health level of older adults [30]. Social media communication is considered an intervention measure to alleviate the loneliness experienced by older adults, achieved by enhancing social support and contact levels, thereby fostering positive responses to emerging technologies [31,32]. Furthermore, the utilization of mobile phones and other media significantly influences disparities in medical care. Increasing the frequency of contact and sustained use of media by older individuals can contribute to unlocking the considerable potential of mobile medical technology in the health care of older individuals [33].

In summary, there is an immediate and practical need to reduce the digital divide among older adults. The willingness of older individuals to embrace mHealth devices, as reflected in surveys, signifies their acceptance of new health technologies and, to a certain extent, their integration into the era of mHealth. Previous research on factors influencing the intention to use mHealth devices among older adults has predominantly centered on understanding the behavioral motivations and mechanisms behind users’ intentions to use, emphasizing the impact of technical and social aspects on actual usage behavior [34]. Research on influencing factors has primarily delved into age, gender, education level, BMI, income, and health status, among other individual aspects [35-37]. However, there is a paucity of studies examining external environmental factors, notably the influence of family and social dynamics, particularly among the older adult population in China. A previous study indicated that family internet access enhances older adults’ cognitive function and increases the frequency of media use [38]. Moreover, family support has been identified as a crucial factor aiding older adults in overcoming barriers to the utilization of mHealth services [39]. Considering the substantial impact of family factors on the proactive health information-seeking behavior of older individuals [40-43], it becomes imperative to delve deeper into the relationship between family health, media use behavior, and the older individual’s intention to use mHealth devices. Additionally, exploring the mediating role of media use behavior between family health and the older individual’s intention to use mHealth devices is crucial. This comprehensive investigation aims to facilitate the integration of older individuals into the “digital age” starting from the family level, foster the adoption of mHealth in the health care sector, enhance societal healthy aging, and contribute to the realization of the objectives outlined in the “Healthy China 2030 Plan.”

### Objectives

In this study, information pertaining to family health, media use behavior, and the intention to use mHealth devices among older adults was gathered from the Psychology and Behavior Investigation of Chinese Residents (PBICR) study. The primary objective of this study was to examine the impact of family health and media use behavior on the intention of older individuals to use mHealth devices in China. Furthermore, the study aimed to assess whether media use behavior acts as a mediating factor in the relationship between family health and the intention to use mHealth devices among older adults. Drawing upon the insights gained from the literature review, the following hypotheses were formulated: (1) family health has a direct impact on the intention to use mHealth devices among older adults; (2) family health exerts an indirect influence on the intention to use mHealth devices through the mediating factor of media use behavior; in other words, media use behavior serves as a mediator in the relationship between family health and the intention to use mHealth devices.

## Methods

### Study Design and Setting

The data for this study were sourced from the PBICR survey, a comprehensive cross-sectional survey initiated by the Peking University School of Public Health in 2022. The survey encompasses 148 cities spanning 23 provinces, 5 autonomous regions, and 4 municipalities directly under the central government in China. Using a multistage sampling approach, the survey uses a stratified sampling method in cities, districts, counties, and communities, and uses a quota sampling method from the community level down to the individual level.

The survey was carried out by adeptly trained investigators. Electronic questionnaires (developed previously [44]) were distributed directly to the public through one-on-one, face-to-face interactions on-site. Respondents could access the questionnaire by scanning the provided QR code. In situations where face-to-face investigations were impeded due to the constraints of the COVID-19 epidemic, investigators distributed the electronic questionnaire on a one-on-one basis through instant communication tools such as WeChat (Tencent Holdings Ltd.). Additionally, online video investigations were conducted through platforms such as Tencent Meeting (Tencent Holdings Ltd.)and WeChat video [45].

Within the PBICR survey, investigators underwent comprehensive training in sampling methods, research tools, and quality control. Only those investigators who strictly adhered to the trained survey procedures were deemed qualified and eligible to participate in the study. Furthermore, during the data processing phase, 2 researchers were designated to perform logical checks. Questionnaires that did not meet the predetermined screening criteria were excluded, ensuring the quality and reliability of the data. Additionally, in this study, further screening was implemented to eliminate questionnaires completed in an excessively short time, those containing outliers, or those with missing values.

In the 2022 PBICR survey, a total of 23,414 questionnaires were collected. Following logical checks and the elimination of outliers, 21,916 questionnaires were deemed valid. For the purposes of this study, the focus will be confined to the age group of 60 years and above. Consequently, the final sample size included 3712 older adults after sorting.

### Participants

A total of 21,916 questionnaires were collected, with the screening criterion being individuals aged 60 years and above, ensuring the absence of missing data and logic errors. Following a meticulous summary and screening process, 3712 valid survey responses were obtained for analysis in this study.

The inclusion criteria for participants in this study were as follows: (1) age between 18 and 60 years old; (2) possession of the nationality of the People’s Republic of China; (3) status as a Chinese permanent resident with an annual travel time of 1 month or less; (4) willing participation in the study and voluntary completion of the informed consent form; (5) ability to independently complete the questionnaire survey or do so with the assistance of investigators; (6) capacity to comprehend the meaning of each item in the questionnaire.

The exclusion criteria for participants in this study were as follows: (1) individuals with unconsciousness or mental disorders; (2) individuals with cognitive impairment; (3) those currently participating in other similar research projects; and (4) individuals unwilling to collaborate or reluctant to participate in the study.

### Ethics Approval

The study adhered to the principles outlined in the Declaration of Helsinki. Ethical approval for all experimental protocols was granted by the ethics research committees of the Health Culture Research Center of Shaanxi (approval number JKWH-2022-02) and Second Xiangya Hospital of Central South University (approval number 2022-K050). The cover page of the questionnaire provided a clear explanation of the study’s purpose and assured participants of anonymity, confidentiality, and the right to refuse participation. Informed consent was obtained from all participants involved in the study.

The questionnaire cover used in this study provided a detailed explanation of the study’s purpose and ensured participants of anonymity, confidentiality, and the right to refuse participation. All participants were required to voluntarily sign an informed consent form before engaging in the study. While respondents did not directly benefit from the survey, their input contributed to a more comprehensive and systematic understanding of the physical and mental health status of the public. The data from this study will be strictly managed and used in accordance with the Statistics Law of the People’s Republic of China. The research data are intended for academic purposes only, and when the research findings are published, no information about individual participants will be disclosed or adversely affected.

### Measurements

#### General Situation Survey Information

The basic demographic information of the older individuals included gender, age rank, nationality, religion, BMI rank, political status, status of occupation, education level, chronic diseases, and family type (conjugal family, core family, backbone family, and other family).

Family types were defined as follows:

- Conjugal family: a family consisting of only husband and wife.
- Core family: a family consisting of parents and unmarried children.
- Backbone family: a family consisting of parents and married children.
- Other family: other families including joint families, single-parent families, DINK (dual income, no kids) families, and single families.

#### Short-Form of the Family Health Scale

The assessment of family health in this study used the Chinese version of The Short-Form of the Family Health Scale (FHS-SF), developed by Crandall et al [20]. Wang et al [46] introduced the FHS-SF cross-culturally to create a Chinese version as a quantitative tool for evaluating family health issues in China. The scale comprises 10 items, encompassing 4 dimensions: family social and emotional health processes, family health lifestyle, family health resources, and family external social supports. A 5-point Likert scale was used for each item of the FHS-SF, with response options ranging from 1=strongly disagree to 5=strongly agree. Items with negative wording were scored in reverse. The final score on the scale ranged from 10 to 50, where higher scores indicated higher levels of family health. Wang et al [46] reported that the Cronbach α for the FHS-SF was .83. Additionally, the Cronbach α for the 4 subscales ranged from .70 to .90, and the retest reliability of the scale was 0.75.

In our study, the composite reliability values for the 4 dimensions were 0.912, 0.848, 0.781, and 0.806, respectively. All these values surpass the reliability threshold of 0.7. The average variance extracted values for the dimensions were 0.775, 0.736, 0.553, and 0.677, respectively, all of which exceed the threshold of 0.5. The Cronbach α of the FHS-SF was .90, and the factor loadings ranged from 0.73 to 0.90, all within an acceptable range.

#### Media Use Behavior Scale

The frequency of media use in this study was gauged using the Media Use Behavior Scale developed by the PBICR survey of Peking University. The scale encompasses various media channels such as newspapers, radio, television, the internet, and mobile phones. Comprising 6 items related to social contact, self-presentation, social behavior, leisure and entertainment, access to information, and business transactions, the scale uses options that signify the degree of media use frequency, ranging from “1=infrequent” to “5=frequent.” The total score on the scale ranges from 6 to 30, with higher scores indicative of more frequent use of the media [45].

In this study, the composite reliability for the Media Use Behavior Scale was 0.894, and the average variance extracted was 0.585. The Cronbach α for the Media Use Behavior Scale was .89, indicating strong internal consistency. Additionally, the standardized factor loadings obtained from the validation factor analysis were above 0.50, all falling within acceptable limits.

#### Intention to Use mHealth Devices

The intention to use mHealth devices in this study was assessed through subjective evaluations. Participants were required to provide a numerical response ranging from 0 to 100 based on their individual subjective awareness. This formed a continuous variable, where a higher numerical value indicated a stronger intention to use mHealth devices.

### Data Analysis

Continuous variables were assessed for normality using the Kolmogorov-Smirnov test and presented as the median and IQR. Categorical variables were reported in terms of frequency and percentage. Nonparametric methods were used to test the differences in characteristics related to the total score of the intention to use mHealth devices. Specifically, the Mann-Whitney *U* test was used for dichotomous variables, while the Kruskal-Wallis *H* test was used for multicategorical variables. The partial correlation coefficient between family health scores, media use behavior scores, and intention to use mHealth devices scores was calculated using a regression model. Linear regression models were used to assess the association between family health scores and media use behavior/intention to use mHealth devices scores, both with and without adjustment for covariates. The associations between media use behavior and intention to use mHealth devices scores were also examined. The results are reported as coefficients along with 95% CIs. Covariates, determined based on previous studies and general knowledge, were included in the models for adjustment. To examine the mediating role of media use behavior scores in the association between family health scores and intention to use mHealth devices scores, we conducted a Sobel-Goodman Mediation Test. This analysis was performed while controlling for all selected covariates. The significance of the indirect effect, direct effect, and the total effect was determined using the bootstrap algorithm.

All *P* values were 2-sided, with a significance level (α) of .05 used to define statistical significance. The data were analyzed using IBM SPSS Statistics 26 and R version 4.1.3 (R Foundation).

### Subgroup Analysis

Indeed, empirical studies have consistently indicated a positive association between education and health. Individuals with higher levels of education often exhibit a tendency to adopt healthier lifestyles, and their increased income may lead to greater investment in health-related expenses [47]. Furthermore, education is closely linked to varying levels of internet participation. Generally, individuals with higher educational attainment are more likely to use online platforms for accessing health-related information [48]. In diverse educational and cultural backgrounds, individuals may exhibit varying levels of concern regarding health risks, subsequently influencing their acceptance of health care technology [49]. Additionally, preliminary analysis in our study revealed significant differences in the total score of family health across different education levels (*P*<.001). Building on the established influence of education on health behavior and media use, as outlined in the existing literature and supported by our results, this paper intends to analyze education level as a subgroup. The aim is to comprehensively explore the mediating role of media use behavior among older adults with different education levels in the relationship between family health and their intention to use mHealth devices.

## Results

### General Characteristics

A total of 3712 older individuals aged 60 and above participated in this study, with an average age of 69.23 (SD 6.13) years. The majority of older adults (3036/3712, 81.79%) fell within the age range of 60-74 years. Basic demographic data for the 3712 older adult participants are detailed in Table 1. Among them, 1839 were males (49.54%) and 1873 were females (50.46%). The majority identified as Han nationality (3370/3712, 90.79%) and nonreligious (3416/3712, 92.03%), with the majority expressing mass political views (3151/3712, 84.89%). There were noteworthy differences in the willingness to use mHealth devices among older adults with varying political statuses, occupational statuses, and chronic disease conditions (*P*<.001). However, no significant differences were observed in the willingness to use mHealth devices among older adults with different family types (*P*=.97; Table 1).

**Table 1:** Characteristics of respondents (n=3712)^a^.

Grouping of characteristics		All (n=3712), n (%)	The total score of the intention to use mobile health devices, median (IQR)	*P* value
**Gender**				.31
	Male	1839 (49.54)	66.00 (36.00)	
	Female	1873 (50.46)	64.00 (35.00)	
**Age rank (years)**				.45
	60≤age≤74	3036 (81.79)	65.00 (34.00)	
	75≤age≤89	662 (17.83)	64.50 (37.00)	
	Age≥90	14 (0.38)	61.00 (60.00)	
**Nationality**				.08
	Han	3370 (90.79)	64.00 (35.00)	
	Minority	342 (9.21)	68.00 (34.00)	
**Religion**				.01
	No	3416 (92.03)	65.00 (35.00)	
	Yes	296 (7.97)	61.00 (35.00)	
**BMI rank (kg/m^2^)**				.001
	Normal (18.5≤BMI<24)	2355 (63.44)	66.00 (34.00)	
	Underweight (BMI<18.5)	373 (10.05)	60.00 (39.00)	
	Overweight (24≤BMI<28)	841 (22.66)	66.00 (36.00)	
	Obese (BMI≥28)	143 (3.85)	60.00 (38.00)	
**Political status**				<.001
	The masses	3151 (84.89)	64.00 (35.00)	
	Communist Party members	512 (13.79)	69.00 (39.00)	
	Probationary Party members	10 (0.27)	59.00 (36.00)	
	Other parties	39 (1.05)	60.00 (38.00)	
**Occupation status**				<.001
	On the job	98 (2.64)	70.00 (44.00)	
	Retired	2071 (55.79)	67.00 (33.00)	
	Rolling stone	392 (10.56)	68.00 (34.00)	
	Unemployed	74 (1.99)	55.00 (41.00)	
	Job waiting/jobless	1077 (29.01)	60.00 (38.00)	
**Education level**				.004
	Primary school and below	1817 (48.95)	63.00 (37.00)	
	Middle school/vocational school/high school	1426 (38.42)	65.00 (34.00)	
	College and above	469 (12.63)	70.00 (35.00)	
**Chronic disease**				<.001
	No	1552 (41.81)	69.00 (35.00)	
	Yes	2160 (58.19)	62.00 (36.00)	
**Family type**				.97
	Conjugal family	887 (23.90)	64.00 (37.00)	
	Core family	325 (8.76)	64.00 (40.00)	
	Backbone family	1854 (49.95)	65.50 (33.00)	
	Other family	646 (17.40)	65.00 (37.00)	

^a^Median (IQR) was used to describe the continuous variable, whereas n (%) was used to describe the categorical variable.

### Association Analysis

After adjusting for covariates, the intention to use mHealth devices exhibited a positive correlation with the total score of family health (*r*=0.077, *P*<.001) and the media use behavior score (*r*=0.178, *P*<.001). Additionally, the total score of family health was positively correlated with the media use behavior score (*r*=0.079, *P*<.001; Table 2).

**Table 2:** Partial correlation coefficients (r) among family health, media use behavior, and intention to use mobile health devices^a^.

Correlations	Family health total score, *r* (*P* value)	Media use behavior scores, *r* (*P* value)	Intention to use mobile health devices, *r* (*P* value)
Family health total score	N/A^b^	N/A	N/A
Media use behavior scores	0.079 (<.001)	N/A	N/A
Intention to use mobile health devices	0.077 (<.001)	0.178 (<.001)	N/A

^a^The model was adjusted for various covariates, including religion, BMI rank, political status, occupational status, education degree, and chronic diseases. Variables achieved statistical significance at *P*≤.05.

^b^N/A: not applicable.

### Relationship Between Family Health and Media Use Behavior Score/Intention to Use mHealth Devices

In the linear regression models before adjustment, the 4 dimensions of family health (ie, family socialization, family healthy lifestyle, family health resources, and family external social support) and the total score were significantly (*P*<.001) associated with media use behavior. Moreover, they were significantly (*P*<.001) related to the intention to use mHealth devices, except for family health resources (*P=*.15). After adjusting for gender and age rank, as well as political status, nationality, religion, BMI rank, occupation status, education level, family type, and chronic diseases, all dimensions remained statistically significant (*P*<.001) except for family health resources (*P=*.29; Table 3).

**Table 3:** Linear regression analysis for media use behavior and intention to use mobile health devices associated with family health^a^.

Analysis element	Media use behavior scores						Intention to use mobile health devices				
	Unadjusted			Adjusted			Unadjusted		Adjusted		
	β (95% CI)	*P* value	β (95% CI)		*P* value	β (95% CI)		*P* value		β (95% CI)	*P* value
Family social	.42 (0.35 to 0.49)	<.001	.32 (0.25 to 0.39)		<.001	.88 (0.56 to 1.20)		<.001		.84 (0.52 to 1.16)	<.001
Family healthy lifestyle	.55 (0.45 to 0.66)	<.001	.45 (0.36 to 0.55)		<.001	1.28 (0.81 to 1.74)		<.001		1.28 (0.81 to 1.75)	<.001
Family health resources	–.26 (–0.32 to –0.20)	<.001	–.25 (–0.31 to –0.20)		<.001	–.19 (–0.46 to 0.07)		.15		–.15 (–0.41 to 0.12)	.29
Family external social supports	.61 (0.15 to 0.71)	<.001	.53 (0.43 to 0.62)		<.001	1.43 (0.98 to 1.88)		<.001		1.45 (1.00 to 1.89)	<.001
Total	.09 (0.07 to 0.12)	<.001	.07 (0.04 to 1.00)		<.001	.30 (0.17 to 0.42)		<.001		.30 (0.18 to 0.43)	<.001

^a^Data were adjusted for gender and age rank, political status, nation, religion, BMI rank, status of occupation, education degree, family type, and chronic diseases.

### Relationship Between Media Use Behavior Score and Intention to Use mHealth Devices

In the linear regression models before adjustment, media use behavior was significantly (*P*<.001) associated with the intention to use mHealth devices. After adjusting for gender and age rank, as well as political status, nationality, religion, BMI rank, occupation status, education level, family type, and chronic diseases, the association remained statistically significant (*P*<.001; Table 4).

**Table 4:** Linear regression analysis for intention to use mobile health devices associated with media use behavior^a^.

Analysis element	Intention to use mobile health devices				
	Unadjusted			Adjusted	
	β (95% CI)	*P* value	β (95% CI)		*P* value
Media use behavior score	.94 (0.80-1.08)	<.001	.84 (0.69-0.99)		<.001

^a^Data were adjusted for gender and age rank, political status, nation, religion, BMI rank, status of occupation, education degree, family type, and chronic diseases.

### Mediation Analysis

**Figure 1:**
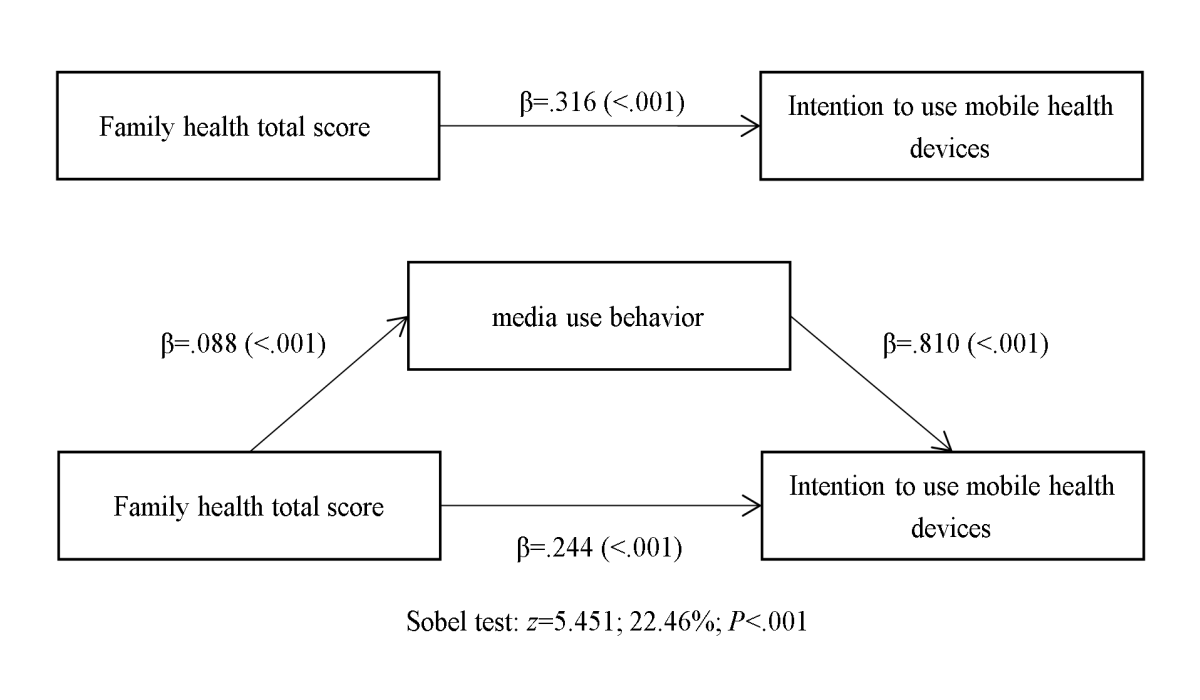
Mediation analysis.

The family health total score demonstrated a positive association with the intention to use mHealth devices among older adults. Mediation analysis, including media use behavior, revealed that the relationship between the total score of family health and the intention to use mHealth devices was mediated through media use behavior. In this study, media use behavior partially mediated the association between family health and the intention to use mHealth devices. The mediating variable accounted for nearly a quarter (22.46/100) of the association when adjusting for covariates. The total score of family health was associated with media use behavior (β=.088, *P*<.001) and intention to use mHealth devices (β=.244, *P*<.001). Additionally, media use behavior was linked to the intention to use mHealth devices (β=.810, *P*<.001). The final mediation models depicting the independent variable (total score of family health), the mediating variable (media usage behavior), and the dependent variable (intention to use mHealth devices) are illustrated in Figure 1.

The 4 dimensions of family health were positively associated with the use of mHealth devices among older adults, except for the dimension of family health resources, which had a nonsignificant association (*P=*.72). The mediation analysis involving media use behavior indicated that the direct and total effects of family health resources were not significant (*P*=.72 and *P*=.20, respectively). Media use behavior acted as a full mediator when adjusting for covariates. Media use behavior partially mediated the relationship between family social, family healthy lifestyle, family external social support, and the intention to use mHealth devices, with mediating effects of 35.18/100, 31.78/100, and 31.33/100, respectively, under adjusted covariates (Table 5).

**Table 5:** Mediation analysis.

Analysis	Family social, β (*P* value)	Family healthy lifestyle, β (*P* value)	Family health resources, β (*P* value)	Family external social supports, β (*P* value)	Total, β (*P* value)
α coefficient	.401 (<.001)	.545 (<.001)	–.284 (<.001)	.616 (<.001)	.088 (<.001)
β coefficient	.790 (<.001)	.789 (<.001)	.844 (<.001)	.770 (<.001)	.810 (<.001)
Indirect effect	.317 (<.001)	.431 (.008)	–.239 (<.001)	.475 (<.001)	.071 (<.001)
Direct effect	.584 (<.001)	.925 (<.001)	.048 (.72)	1.042 (<.001)	.244 (<.001)
Total effect	.901 (<.001)	1.356 (<.001)	–.191(.20)	1.516 (<.001)	.316 (<.001)
The proportion of total effect that is mediated, n/N	35.18/100	31.78/100	125.13/100	31.33/100	22.46/100
Sobel test^a,b^	7.754 (<.001)	7.497 (<.001)	–7.284 (<.001)	–7.284 (<.001)	5.451 (<.001)

^a^The Sobel-Goodman Mediation Test was applied in adjusted models for religion, BMI rank, political status, occupation status, education level, and chronic diseases.

^b^The Sobel test was used to assess the hypothesis that the indirect role was equal to 0, adjusting for covariates such as religion, BMI rank, political status, occupation status, education level, and chronic diseases. Values reach statistical significance at *P*≤.05.

### Subgroup Analysis

Subgroup analyses based on education degrees are presented in Table 6. Among the older adult population with primary school education and below, media use behavior showed no mediating effect between the total score of family health and the intention to use mHealth devices (*z*=–0.942; indirect effect=–0.019, *P*=.35; direct effect=0.252, *P*=.007). Additionally, the mediating effect of media use behavior between family healthy lifestyles and the intention to use mHealth devices was not significant (*z*=1.953, *P*=.052). Media use behavior fully mediated the association between family health resources scores and intention to use mHealth devices scores in different education degrees among the older adult population: primary school and below degree older adult population (*z*=–5.832; indirect effect=–0.331, *P*<.001; direct effect=0.218, *P=*.29), middle school/vocational school/high school degree older adult population (*z*=–3.439; indirect effect=–0.136, *P*<.001; direct effect=–0.066, *P*=.76), and college and above degree older adult population (*z*=–2.516; indirect effect=–0.212, *P=*.01; direct effect=0.026, *P*=.93).

**Table 6:** Subgroup analysis of education degree of mediation models for family health associated with intention to use mobile health devices mediated by media use in older adults.

Education and parameters			Family social, β (*P* value)		Family healthy lifestyle, β (*P* value)		Family health resources, β (*P* value)		Family external social supports, β (*P* value)		Total, β (*P* value)
**Primary school and below**											
	α coefficient	.099 (.04)		.140 (.046)		–.302 (<.001)		.246 (<.001)		–.018 (.34)	
	β coefficient	1.059 (<.001)		1.057 (<.001)		1.093 (<.001)		1.046 (<.001)		1.078 (<.001)	
	Indirect effect	.105 (.04)		.148 (.05)		–.331 (<.001)		.257 (<.001)		–.019 (.35)	
	Direct effect	.489 (.04)		.904 (.01)		.218 (.29)		.808 (<.05)		.252 (.007)	
	Total effect	.593 (.01)		1.052 (.003)		–.112 (.59)		1.065 (<.01)		.232 (.015)	
	Proportion of total effect that is mediated, n/N	17.71/100		14.07/100		295.54/100		24.13/100		–8.19/100	
	Sobel test^a^	2.052 (.04)		1.953 (.052)		–5.832 (<.001)		3.485 (<.001)		–.942 (.34)	
**Middle school/vocational school/high school**											
	α coefficient	.508 (<.001)		.718 (<.001)		–.250 (<.001)		.706 (<.001)		.123 (<.001)	
	β coefficient	.484 (<.001)		.477 (<.001)		.541 (<.001)		.458 (<.001)		.508 (<.001)	
	Indirect effect	.246 (<.001)		.343 (<.001)		–.136 (<.001)		.323 (<.001)		.063 (<.001)	
	Direct effect	.598 (.03)		.992 (.01)		–.066 (.76)		1.200 (.002)		.227 (.03)	
	Total effect	.845 (.002)		1.334 (<.001)		–.201 (.35)		1.524 (<.001)		.290 (.005)	
	Proportion of total effect that is mediated, n/N	29.11/100		25.71/100		67.66/100		21.19/100		21.72/100	
	Sobel test^a^	3.624 (<.001)		3.563 (<.001)		–3.439 (<.001)		3.455 (<.001)		3.375 (<.001)	
**College and above**											
	α coefficient	.589 (<.001)		.713 (<.001)		–.218 (.004)		1.057 (<.001)		.175 (<.001)	
	β coefficient	.808 (<.001)		.874 (<.001)		.974 (<.001)		.737 (<.001)		.864 (<.001)	
	Indirect effect	.476 (<.001)		.624 (<.001)		–.212 (.01)		.779 (<.001)		.151 (<.001)	
	Direct effect	1.260 (.003)		1.183 (.04)		.026 (.93)		1.926 (<.001)		.425 (.009)	
	Total effect	1.736 (<.001)		1.807 (.002)		–.186 (.56)		2.704 (<.001)		.576 (<.001)	
	Proportion of total effect that is mediated, n/N	27.42/100		34.53/100		1139.78/100		28.81/100		26.22/100	
	Sobel test^a^	3.409 (<.001)		3.428 (<.001)		–2.516 (.01)		3.310 (<.001)		3.193 (.001)	

^a^The Sobel-Goodman Mediation Test was applied in adjusted models for religion, BMI rank, political status, status of occupation, and chronic diseases.

## Discussion

### Principal Findings

Previous studies have consistently demonstrated that family factors play a crucial role in influencing the frequency of media use and the acceptance of mHealth among older adults [50]. The findings of our study further confirm that family health positively contributes to increasing the willingness of older adults to use mHealth devices. Additionally, a high frequency of media use behavior emerges as a significant driver for the utilization of mHealth devices, a behavior that is profoundly influenced by the state of family health. The results align with previous research on the digital divide among older adults, indicating that those with higher family health scores tend to engage in more frequent media contact behaviors. This heightened connectivity to the internet makes them more adaptable to a big data–based mHealth environment, fostering a greater willingness to use mHealth devices. Before conducting the mediation analysis, the study also observed, through univariate analysis, that older individuals over 90 years and those who were unemployed exhibited a lower willingness to use mobile medical devices. The results confirm the existence of differences in the digital divide among age groups, especially with older age groups experiencing inequalities in social and economic support [51,52]. These disparities may further impact their access to and utilization of media devices.

In addition to the descriptive findings, this study delves into the intricate relationship between family health and the willingness to use mHealth devices, uncovering the mediating role of media use behavior. Primarily, the study supports the positive impact of media use behavior, which partially mediates the influence of overall family health levels on the intention to use mHealth devices. Furthermore, the results indicate that media use behavior serves as a fully mediating variable in the dimension of family health resources. In essence, the findings suggest that older adults lacking family health resources completely lose their willingness to use mHealth devices, primarily due to their challenges in accessing or using media. This underscores the crucial role of family health resources in integrating older adults into the internet sphere and enabling them to benefit from mHealth technology. The study emphasizes the practical importance of addressing resource-related health inequities, with financial support from the family being identified as a critical factor in the daily lives of seniors [52]. To address the imbalance in the distribution of resources among families in different regions at the societal level, it is crucial for the government to assist socioeconomically disadvantaged older adults in gaining greater access to various devices. This can be achieved through economic empowerment initiatives and the development of policies aimed at bridging the digital divide [53].

Building upon the crucial role of media contacts in linking family health resources and the willingness to use mHealth devices among the older population, there is an opportunity to further motivate the desire for mHealth device usage. Leveraging the positive influence of family health resources to increase the frequency of media exposure can enhance the motivation of older individuals. Effective communication within the family emerges as a catalyst for improving the technology literacy and information-seeking skills of older adults [16]. Family members play a crucial role in supporting seniors to build confidence in using internet technology while alleviating their anxiety and fear of new technologies. Encouraging older adults to adapt and learn information technology, such as WeChat and health-related mobile apps, through straightforward and repeated demonstrations can be an effective strategy [54]. Additionally, family support may help mitigate the economic challenges associated with using health care services by influencing older adults’ subjective perceptions of financial accessibility [55]. To address financial challenges and enhance older adults’ access to technology, a comprehensive approach can be adopted. This involves leveraging both the financial support within the family and external economic resources. Encouraging family members to provide suitable financial assistance to each other, coupled with ensuring stable financial security for older individuals, can be achieved by gradually increasing pensions for retirees. This approach aims to augment the purchasing power of older adults, enabling them to acquire media devices and enhancing their ability to use technological devices in the health care sector to a greater extent.

The subgroup analysis further indicated that media use behavior did not mediate the relationship between the total family health score and the intention to use mHealth devices among older adults with primary school education or below. However, it did partially mediate the association among those with primary school education and above, aligning with the study hypothesis. Given that the older adult population with low education levels may experience relatively weak cognitive function and lack personal health literacy [56,57], the mechanisms by which they are influenced by family, social, and economic environments in the acceptance of new health technologies become more intricate. Conversely, older adults with a high school education or higher often perceive themselves as having an above-average ability to learn, making them less uncomfortable with the changing social environment brought about by technological developments [58]. Moreover, older individuals with limited education often lack access to information technology education or the ability to operate mobile devices [59]. For these individuals, exposure to media devices or mHealth devices is relatively homogeneous. Consequently, they may lack a progressive transition from regular media contact behaviors to the use of mHealth devices.

Disparities in internet participation levels due to education constitute a significant barrier hindering older adults from using media devices to access the mHealth era. To bridge the “digital divide” and enhance the effective use of mHealth devices among older individuals, it is imperative to consider implementing relevant education measures. These measures can focus on improving their ability to use smart technology, thus empowering them to navigate and benefit from the advancements in health care technology. In alignment with the comprehensive “Smart Senior Care” action plan in China [60], communities can implement health education initiatives through a blend of technology-supported learning and traditional lectures. For instance, using touchscreen tablets for courses on healthy diet and nutrition guidance can enhance the older individual’s interest in the internet while imparting essential health and hygiene knowledge [61]. This approach serves to bridge the transition from traditional modes of access to mobile health care. Adopting adaptive behaviors and learning strategies can further enhance the efficiency and effectiveness of mobile health care apps [62]. In the mHealth era, the design of mHealth devices should be tailored to the cognitive abilities and mindset of older individuals. Full consideration should be given to their eHealth literacy, incorporating improvements in usability, emphasizing the responsiveness of operations, and integrating monitoring functions that align with the physical activities of older individuals [63]. Such considerations aim to enhance the overall satisfaction of older individuals with mobile health care apps [64]. Moreover, due to prevailing stereotypes about older people, digital platforms often harbor ageist mechanisms that categorize them as users uninterested in technology [65]. This results in an unfavorable digital environment for older individuals. In general, the development and application of internet technology must not overlook the realistic capacity and objective demands of older individuals [66]. Digital platforms should strive to create more inclusive algorithms and use statistical models of social digital media practices that cater to all literacy levels [65]. This may involve reducing complex and lengthy text that is difficult to understand, avoiding in-depth and complex hierarchical options, and adopting simple page designs [67] to mitigate the impact of technological differences on the accessibility of digital health care for older adults.

### Strength and Limitations

This study contributes significantly to the existing literature by evaluating the connection between family health, media use behavior, and the intention to use mHealth devices among older adults, using cross-sectional data from the PBICR survey. The findings of this study support our hypothesis that media use behavior serves as a mediator between family health status and the intention to use mHealth devices among older adults. Furthermore, a subgroup analysis based on education level revealed that the impact of family health on the willingness to use mHealth devices through media use behavior was not significant among older adults with lower education levels, indicating a nuanced mechanism at play. All of the aforementioned studies contribute to the body of research on the digital divide among older individuals.

Despite comprehensive consideration, the results of this study have several limitations. First, due to the exploratory cross-sectional design, no causal inferences can be drawn. Second, the majority of seniors included in this study were in the young-old age group (60 to 74 years old), lacking representation of the entire age spectrum of older adults and potentially neglecting variations in social background associated with age factors. Third, the results obtained in this study may be influenced by economic factors and psychological variables. As mHealth devices represent an evolving component of the health system, their development trajectory is still undergoing exploration. It is possible that various latent factors influencing the relationship between family health, media use behavior, and the intention to use mHealth devices are yet to be uncovered.

### Conclusions

In conclusion, this study highlights the substantial impact of family health and media use behavior on the intention of older adults to use mHealth devices. Media use behavior acts as a mediator in the relationship between family health and the intention to use mHealth devices, with more intricate dynamics observed among older adults with lower educational levels. These findings emphasize that robust family health, particularly sufficient family health resources, plays a crucial role in enhancing the media engagement of older individuals, ultimately fostering their interest in embracing mHealth devices. The insights from this work provide valuable recommendations for bridging the gap in digital health adoption among older adults. Furthermore, encouraging teaching by family members can create a supportive environment for seniors to embrace mobile technology, while financial support can enhance their accessibility to health-related mobile devices. Additionally, developing age-specific digital education programs and media products tailored to the needs and preferences of older individuals can contribute to overcoming technological barriers and fostering a positive digital experience for older adults in the realm of mobile health care. These strategies align with the goal of promoting inclusive and user-friendly digital solutions for seniors, ensuring they can benefit from advancements in health technology.

## Abbreviations

DINK: dual income, no kids
FHS-SF: Short-Form of the Family Health Scale
mHealth: mobile health
PBICR: Psychology and Behavior Investigation of Chinese Residents

## Data Availability

The data sets generated and analyzed during this study are not publicly available because the data still need to be used for other research but are available from the corresponding author on reasonable request.
